# Health Needs of Trans and Gender Diverse Adults in Australia: A Qualitative Analysis of a National Community Survey

**DOI:** 10.3390/ijerph16245088

**Published:** 2019-12-13

**Authors:** Sav Zwickl, Alex Wong, Ingrid Bretherton, Max Rainier, Daria Chetcuti, Jeffrey D. Zajac, Ada S. Cheung

**Affiliations:** 1 Trans Medical Research Group, Department of Medicine (Austin Health), The University of Melbourne, Heidelberg, Victoria 3084, Australia; sav.zwickl@unimelb.edu.au (S.Z.); fwong@deakin.edu.au (A.W.); ibretherton@student.unimelb.edu.au (I.B.); maxatlas@gmx.com (M.R.); daria.chetcuti@unimelb.edu.au (D.C.); j.zajac@unimelb.edu.au (J.D.Z.); 2 Department of Endocrinology, Austin Health, Heidelberg, Victoria 3084, Australia

**Keywords:** transgender persons, gender identity, health services, health services needs and demand, health services for transgender persons

## Abstract

There is an increasing demand for trans and gender diverse (TGD) health services worldwide. Given the unique and diverse healthcare needs of the TGD community, best practice TGD health services should be community-led. We aimed to understand the healthcare needs of a broad group of TGD Australians, how health professionals could better support TGD people, and gain an understanding of TGD-related research priorities. An anonymous online survey received 928 eligible responses from TGD Australian adults. This paper focuses on three questions out of that survey that allowed for free-text responses. The data were qualitatively coded, and overarching themes were identified for each question. Better training for healthcare professionals and more accessible transgender healthcare were the most commonly reported healthcare needs of participants. Findings highlight a pressing need for better training for healthcare professionals in transgender healthcare. In order to meet the demand for TGD health services, more gender services are needed, and in time, mainstreaming health services in primary care will likely improve accessibility. Evaluation of training strategies and further research into optimal models of TGD care are needed; however, until further data is available, views of the TGD community should guide research priorities and the TGD health service delivery.

## 1. Introduction

With increasing visibility of trans and gender diverse (TGD) people and increasing demand for TGD health services worldwide [1,2], attempts are being made to design appropriate health services. Correspondingly, there has been an exponential rise in the number of research studies published in TGD health [3]. The TGD community has unique and diverse healthcare needs that are frequently coupled with societal discrimination and stigma, which may impact upon the trust of health professionals as well as overall health and well-being.

Best practice TGD health services should be community-led and co-designed with the TGD community [4]. Previous small surveys have suggested that increased education of medical practitioners was needed to better engage with gender diverse clients [5]. However, the TGD community is diverse, and small, focused consumer advisory groups may not necessarily capture the views of the broader community. In order to guide policy direction, the design of health services and relevant research areas for the TGD community, we aimed to understand the healthcare needs of a broad group of TGD Australians, how health professionals could better care and support TGD people and gain an understanding of community views on TGD-related medical research priorities.

## 2. Materials and Methods

This anonymous online survey of TGD Australian adults was designed to provide a platform for the TGD community to voice their healthcare needs and priorities. Purposeful, criterion-specific sampling was used to recruit from this minority population. Inclusion criteria were assessed via three screening questions: a) resident of Australia; b) identification as trans or gender diverse or had previously identified as such in the past; c) aged over 18 years.

Participants were recruited for the survey through a post on the Trans Medical Research Facebook page. This post was shared by 275 individuals and online transgender support groups. The survey was also promoted at several LGBTIQA+ events in Sydney and Melbourne, Australia. The survey remained anonymous and as such, written informed consent was not obtained; however, continuation with the survey implied informed consent. Individuals were eligible to complete the survey on one occasion only, and duplicate responses from the same Internet Protocol (IP) address were excluded.

The study was approved by the Austin Health Human Research and Ethics Committee (HREC/17/Austin/372). The online platform SurveyMonkey (SurveyMonkey Inc. San Mateo, California, USA) was used to design and collect responses to the survey. The survey was available online to respondents between 1st September 2017 and 31st January 2018. Questions in the survey covered sociodemographic and clinical data and participants were asked to report past medical diagnoses of various conditions and access to various types of health care providers were determined (reported elsewhere). This paper selected three questions out of the collected data set, which allowed for open free-text responses for qualitative analysis. These were; 1) How do you think healthcare professionals can better support you? 2) What do you think are the two most important issues for your health, i.e., if you could improve your health right now, what would you do? 3) Are there any areas of trans-related medical research that you would like addressed?

The responses from all participants to each of the three survey questions were collated into three data transcripts. One of the researchers (AW) coded the participant responses one-by-one using NVivo qualitative data analysis software version 12 (QSR International Pty Ltd., Doncaster, Australia). Codes were developed from the data during the coding process (i.e., codes were not previously established). A second researcher (SZ) then independently coded the data against these codes and queries and discrepancies in the interpretation of the codes were discussed and resolved. Using NVivo, a coding comparison query was performed to determine the degree of agreement between the two coders. This comparison calculated a Cohen’s Kappa value for each code’s content. The average Cohen’s Kappa value across the different codes for each survey question was calculated, with each code given equal weight.

## 3. Results

A total of 964 responses to the survey were obtained; however, after excluding participants who did not identify as trans or gender diverse, those not living in Australia, and duplicate responses, there was a total of 928 eligible responses to the survey. For the three questions of focus in this analysis, 763 responses were received for questions 1 and 2, (“How do you think healthcare professionals can better support you?” and “What do you think are the two most important issues for your health, i.e., if you could improve your health right now, what would you do?”) and 641 responses were received for question 3 (“Are there any areas of trans-related medical research that you would like addressed?”).

Responses were received from all States and Territories in Australia. In brief, 56% of respondents were birth-assigned females, 43% birth-assigned males, and 1% stated they were intersex. A total of 37% identified as trans female, trans feminine, trans woman or female, 36% identified as trans male, trans masculine, trans man or male, and 27% had non-binary gender identities. The median age of participants was 28 (IQR 23–39). Participants were generally well-engaged with the Australian health care system, with 80% having a regular primary care doctor.

The data from the first question “How do you think healthcare professionals can better support you?” was encoded to 7 codes, which were then separated into two overarching themes; “Provide Accessible Healthcare” and “Training and Professional Development” (see Table 1). The average level of agreement between the two coders, across all codes for the first question, was high, with an average Kappa coefficient of 0.74.

The data from the second question “What do you think are the two most important issues for your health, i.e., if you could improve your health right now, what would you do?” was encoded to 11 codes. Ten of the codes were separated into three overarching themes; “Accessibility and Training for Healthcare Professionals”, “Trans-related medical intervention”, and “Health, Wellbeing and Support” (see Table 2). One of the codes included neutral and unclassifiable responses and was not included under any of the themes (1.7% of responses). The average level of agreement between the two coders, across all codes for the second question, was high, with an average Kappa coefficient of 0.89.

For the third question, “Are there any areas of trans-related medical research that you would like addressed?”, the data were encoded to 10 codes. Nine of the codes were separated into three overarching themes; “Trans Medical Advancements”, “Accessibility and Standards of Care”, and “Greater Breadth of Research and Understanding” (see Table 3). One of the codes included responses such as “don’t know” and “nothing” and was not included under any of the themes (15.7%). The average level of agreement between the two coders, across all codes for the third question, was high, with an average Kappa coefficient of 0.89.

### 3.1. Support Required from Healthcare Professionals: Question 1 “How do You Think Healthcare Professionals can Better Support You?”

#### 3.1.1. Theme 1: Training and Professional Development for Health Professionals

Almost half of participants (44.8%) indicated that knowledge around transgender issues is generally lacking and that greater training of medical professionals around TGD health issues is paramount.

General knowledge of trans* patient care and the medical needs/concerns of transgender patients seems to be extremely lacking amongst doctors. More education is needed.

*Healthcare professionals need to access education on gender identity. The biggest barriers at the moment are misunderstanding and lack of knowledge.*

*From previous experiences I think better education regarding gender diverse healthcare is needed. Ideally it would start in medical school.*

The lack of knowledge meant that many TGD people felt that to have their healthcare needs met, they had to attend a specialist doctor or clinic, which often came with long wait times for appointments and high costs.

*Better education. I have to go to an LGBT clinic just because GPs are ignorant.*

*More awareness of trans issues and treatments. Currently only doctors and surgeons with proven experience in treating trans patients seem to know what to do with us.*

Correspondingly, in addition to an increase in training, just over one in five of the participants (21.4%) reported improvements in professionalism as key. Three main issues around professionalism were raised by participants: the use of correct name and pronouns; inappropriate and irrelevant questions about their transgender experience, and incorrect focus on their TGD status as a cause of any presenting mental and medical health issues.

*Not assume my gender, not laugh at me when I say I need a certain type of medical care, not ask me questions about my transgender experience/status that is irrelevant to the current concern/procedure, ask me my pronouns, use them correctly.*

*If they could stop being convinced that being trans means that my unrelated physical disability is actually just me being crazy/anxious/depressed/psychotic/malingering, that would be excellent. I want my healthcare professionals to use my correct name and pronouns above everything else. They don’t need to address my trans identity unless it is directly relevant to whatever I am seeing them for.*

Other responses to the question “*How do you think healthcare professionals can better support you?”* included provision of reliable health information such as treatment pathways, greater communication with the TGD community as well as a desire for more guidelines, research, and advocacy.

*Flag somehow that they are trans-friendly/queer-friendly, for example, with a note on their website or a sign or sticker, that would be really helpful in helping me figure out which healthcare professionals are safe.*

Ideally healthcare professionals will let the patient lead the discussion regarding what they need. For example, not forcing a trans* person to follow any one medical path for transition.

*All registered GP’s should be sent a pamphlet on the current laws and literature about trans people. And the pathway that they can help their trans patient get onto, hormones, surgery, etc.*

Less than 5% of the participants responded to this question, by reporting they had only had positive experiences with healthcare professionals. A willingness to listen, learn, and respect a person’s name and pronouns were sited as integral to feeling supported.

*I am lucky to have a number of GPs at the clinic I attend who are very supportive of patient-led care and who go out of their way to ensure any additional practitioners I see will be trans-friendly. I wish all healthcare professionals would do this.*

*A willingness to try and understand the thoughts and feels we go through when they haven’t encountered a trans person before. My current healthcare professionals have done this for me and that has made me stop seeing others to see them.*

#### 3.1.2. Theme 2: Provide Accessible Care

Improving the accessibility of medical interventions to aid gender transition was a major theme of responses to how health professionals could assist TGD people. An integral issue reported was ‘gatekeeping’; referencing a need to undertake multiple psychiatric assessments in order to be deemed suitable for hormone treatments or surgery, which often delayed treatment and negatively impacted mental health.

*I’ve only ever been hurt by these gatekeepers. They have never saved me from a mistake, they have only gotten in my way, delayed access to important interventions, and sometimes abusing their total monopoly over my access to health care*

*Stop being hate keepers, making us prove ourselves and meeting your notions of trans and who is deserving of support. Instead, collaborate with us and support us in our health and well being aspirations.*

Out-of-pocket expenses for treatments, particularly gender-affirming surgery, was frequently reported to be prohibitive, yet such treatments were described as vital to improve mental and general wellbeing.

*I think the access to and cost to trans healthcare should be within reach of all trans Australians.*

*Make it more cost effective. Most of us need to get loans or tap into super to pay for surgery and that puts a lot of monetary pressure onto us at a time when we’re at our most vulnerable.*

*Help to make surgery be more affordable. I really want to have genital surgery with the rod implant. But with a price over $90000 that’s not really possible for your average person. Its not just a cosmetic procedure as they say. It is mentally affecting my life in a big way.*

Long wait times and geographical accessibility were also raised as issues to be addressed by healthcare professionals, as well as the lack of availability of certain treatments in Australia.

*Have more professionals who specialise in the area so wait times aren’t so long.*

*Easier access to support. I shouldnt have to travel to a capital city to get gender support.*

*Better pathways towards gender confirming surgeries provided within this country instead of forcing folks to seek the help they NEED outside of the country and putting themselves at risk to do so.*

### 3.2. Self-Perceived Health Issues: Question 2 “What do You Think are the Two Most Important Issues for Your Health, i.e., If You Could Improve Your Health Right Now, What Would You Do?”

#### 3.2.1. Theme 1: Trans and Related Medical Intervention

Transgender related medical interventions were reported to be highly important to the healthcare needs of over one third (36%) of the transgender participants. Specifically, the most common interventions included a range of surgeries, post-surgery support, hormone replacement therapy, speech training, and hair removal.

*Top surgery for better confidence and all-round positive outlook on life.*

*Better post operative support regardless of where you had surgery.*

*Chest/respiratory and back pain issues from long-term binding that has caused damage to my chest and back. I would need top survey ASAP to stop the damage but the waiting list is over a year long, but then my chest could become even more damaged.*

#### 3.2.2. Theme 2: Mental and General Wellbeing

General wellbeing was reported as a key healthcare need by about one third (32.2%) of the participants. This included quality of sleep, exercise and fitness, and a healthier diet.

*Get more sleep and better sleep.*

*Access to post-transition support eg fitness and health (eating disorders and weight gain).*

Some participants reported barriers to achieving their general wellbeing needs, such as difficulty finding dieticians and exercising environments that are affirming and not discriminatory against TGD people.

*Find a place I feel comfortable exercising; public gyms are intimidating due to my trans status.*

*Be able to find exercise classes that are run by or for LGBT people so I feel comfortable and not worse about myself.*

Mental wellbeing concerns were raised as an important healthcare need by almost one-third of participants (31.6%). This included several references to self-harm and suicidal ideation, and a desire to reduce stress, depression, anxiety and other mental health issues, in order to gain a more positive outlook on life.

*Self-harm and almost constant suicidal thoughts.*

*How to deal with stress, which in general would help me with anxiety and dysphoria also.*

Having access to affordable mental health professionals, with training and experience with TGD people was also reported as a self-perceived health issue.

*Accessible psychological support. Currently psychs (more so for specialist trans care) are unavailable or are inaccessible for price.*

*Regular support from a trained, knowledgable psychologist, not just limited to the few sessions via a mental health plan.*

Additionally, 15.1% of participants reported that other medical issues, such as chronic pain, were of key importance. Other self-perceived health issues included a need to reduce social discrimination of transgender people, an increase in social support, housing, employment and family issues, and addressing substance use.

*Broader understanding and acceptance of tran speople in the general community would make my mental and physical health improve tremendously.*

*My fear of other people in public places and lack of understanding by others has a huge effect on my anxiety and depression.*

*Change jobs to somewhere I could be open about my gender identity to improve my mental health.*

#### 3.2.3. Theme 3: Accessibility and Education

Aligned with responses to necessary support required from health professionals, almost one in four participants (22.5%) reported that access to gender-affirming healthcare was one of their most important health issues, including ‘gatekeeping’, the lack of financially accessible specialists, mental health support, and access to gender-affirming medical interventions.

*Surgery (top surgery) would greatly improve both my physical and mental health, however, cost makes this inaccessible.*

*Access a speech pathologist (currently prohibitively expensive); switch from Spironolactone to GnRH analogues (also prohibitively expensive).*

*Access to a psychologist who is fully skilled, aware, and trained to understand both my identities as a nonbinary and demisexual person, as well as fully covered financially.*

In terms of more generalised access to healthcare as a transgender person, discrimination was seen as a central health issue.

*Be able to access medical and psychological services without discrimination, harassment, verbal abuse, or be refused care because I’m trans.*

Like with the first survey question, education for health professionals around transgender experiences and transgender healthcare was reported as a priority. Other keys issues included a desire for greater community building and information for the TGD community, with a number of participants describing difficulting finding trans-related healthcare information.

*Better access to reliable information about what services are available and where to find them.*

*I want to get on hormone therapy but literally have no idea how and who to ask.*

### 3.3. Trans-Related Medical Research Topics: Question 3 “Are There Any Areas of Trans-Related Medical Research That You Would Like Addressed?”

#### 3.3.1. Theme 1: Medical Advancements

Over a quarter of participants (27%) indicated that hormone effects and risks was an important area of TGD medical research.

*I think there should be more research on the long-term effects of hormone treatment and whether there are any risks involved in taking hormones (e.g., increased risk for some cancers).*

*I’d also like to know more about the long term risks of testosterone. Do I need a hysterectomy after 5 years? How many of my health issues can be explained by being on testosterone for 6 years?*

Greater research into gender-affirming surgical techniques was desired by 18.7% of participants. In particular, improvements in bottom surgery techniques and availability were commonly mentioned.

*Australia could do well to increase the training and quality of the surgeons able to provide gender confirming surgeries. Internationally we have seen advancement in trans healthcare that is not being reflected here.*

*Bottom surgery for trans men is inadequate and highly dangerous, more research and improvements need to be done.*

In addition, 6.2% of participants indicated that potential health issues associated with transgender interventions, such as chest binding and genital tucking is an important area of research, while there was also interest in alternative and associated transgender treatments, such as more effective hair removal.

#### 3.3.2. Theme 2: Accessibility and Standards of Care

Access to gender-affirming healthcare was indicated as an important area of research by 10% of participants. This included research into more cost-effective medical interventions and research into better standards of care. Other research areas of community interest included research into diagnosis or treatment guidelines, community support, communication, and the impact of social support on health outcomes in transgender people and the need for more transgender people to conduct research.

*WHAT the options are for transitioning. I STILL can’t find anything definitive.*

*Better guides for how to prescribe hormones.*

#### 3.3.3. Theme 3: Greater Breadth of Research and Understanding

Mental health and neurological indicators were areas of research desired by 15.3% of participants. This included exploration of the correlation between autism spectrum and gender diversity and neurological or other biological indicators for identifying and validating transgender people.

*Correlation between autism spectrum disorders and transgender identity.*

*Further understanding its causes would likely provide greater acceptance in the community so people including trans people can understand and accept that gender identity doesn’t always align with genitals from an early age. I really wish I knew what made me trans it would have made it easier to accept in myself and help my siblings and parent accept me better.*

There were several other research areas desired including non-binary identities and experiences, adolescent issues, aged care and intersex conditions.

*Non binary people, please!! We get forgotten and erased so much.*

*I think there should be more research on the amount of trans people with eating disorders, because in my experience it’s unfortunately common.*

*More research into puberty blockers affects on development to hopefully get rid of some of the stigma and hesitance around its use.*

*Impacts and experiences of stigma, discrimination and also affirmative care and supportive environments.*

Some participants indicated that there should be transgender research across all areas of being TGD.

*Research is totally lacking. I think we’re ignored as a population. The healthcare offered doesn’t seem to be based on good evidence and it’s like we’re medical research subjects undergoing experimental treatment. Nobody knows what they’re doing.*

## 4. Discussion

This large qualitative survey provides an in-depth insight into health and research issues of most concern for the Australian TGD community. A need for better training for healthcare professionals in TGD health and providing ready access to hormonal, surgical, and psychological support to aid gender transition for optimal mental and general well-being were the most commonly reported healthcare needs of TGD people.

Whilst Australia has one of the world’s best health systems that provides universal government-funded healthcare in parallel with the private sector [6], there remain many shortcomings. Our findings suggest that TGD health is one area in need of policy direction and resourcing to best support this marginalised community. Basic requirements, including feeling safe and validated, are not being met. Consistent with previous research [5,7,8,9,10,11], transgender participants in this study frequently indicated that current knowledge, experience, and professionalism amongst healthcare professionals around transgender health is poor and that there is a pressing need for better training for health professionals and more research in transgender healthcare. This includes training regarding using the correct name and pronouns, which has been shown to be vitally important in validating TGD experiences [10,12,13,14]. It can be argued that training programs in transgender healthcare would not only improve knowledge and professionalism and reduce discrimination but would address in part, the issue of lack of access to health services and inability to find doctors to provide gender-affirming treatments [15,16].

Training for all clinicians and patient-facing health professionals, including emergency departments and administration and clerical staff, are very much required, and professional societies need to place TGD health as a training priority [10,14,17]. Moreover, more investment in training programs nationally is very much needed, as well as the implementation of trans cultural awareness amongst various health professional degrees and accreditations. This is not limited to Australia—in a US survey of over 400 practising clinicians, 80% indicated that they had never received training regarding the care of TGD patients even though almost 80% have treated a TGD patient [18].

Ability to access and obtain trans and related medical interventions is undoubtedly of major importance to allow an individual’s physical characteristics to align with their gender identity. Accessibility is, however, an ongoing and prevalent issue [19,20,21]. For example, a recent US study showed that 31% of trans individuals were unable to access hormone therapy [20]. In addition, the TGD community also struggled with general well-being and access to basic community services such as safe environments to exercise. Social factors, such as discrimination, housing, employment, and family issues, also play a large role in overall health. These issues are due to larger societal issues and policies need to be implemented to promote inclusivity, enabling safe environments for all. Financial and geographical accessibility, as well as ‘gatekeeping’, need to be addressed and whilst mental health support is very much desired, the cost and time required to undertake assessments and approvals for interventions were barriers. Policies need to be implemented to direct resources and adequately provide services across the country. An example would be utilising telehealth to reach those far away from metropolitan centres. Participants frequently associated lack of accessibility with poorer health outcomes, which is consistent with previous research that suggests the unique healthcare needs of TGD people, coupled with social discrimination and stigmatisation, negatively impacts their overall wellbeing significantly [19]. These findings indicate that there is a need to address issues around transgender healthcare accessibility, such as making medical interventions more financially affordable, increasing regional access and more broadly implementing informed consent for hormone therapy.

While systematic reviews may indicate that there is a substantial amount of literature [3], TGD research has historically been problematic, with the widespread pathologisation of TGD individuals, and there are still many significant gaps in the literature. The community, overall, are supportive of research into improving TGD health outcomes and particularly desired research in hormone effects and surgical techniques. Overall, health service delivery was also a key area of research that should be implemented. Interestingly, despite much debate regarding gender as a social construct [22,23,24], there were views expressed that desired exploration into links between neurodevelopmental conditions and gender as well as genetic or biological indicators for validating transgender people. Research should also focus on less well-understood groups within the TGD community at large, including non-binary experiences, TGD health in aged care, and intersex conditions. There is also a need to develop ethical recommendations and guidelines in the area of transgender health research and presentation of this research, including avoiding the use of disrespectful language and intentional or unintentional misgendering of TGD individuals [25]. Research should be performed to benefit the health of communities, and addressing these broad areas of interest will ensure that research remains relevant to community needs.

There were some limitations to this study inherent in the design. Firstly, the survey was anonymous and self-reported, and data responses to these questions were not measured objectively. While some responses were detailed, many participants provided very short responses that limited inferences. There was also no control sample of non-transgender Australian individuals to use as a comparison between the healthcare experiences of transgender individuals and the wider population. There was also an over-representation of younger individuals and under-representation of older individuals, and this is likely related to online recruitment methods. Given the rapidly changing expression and understanding of gender, it is likely that the needs and wants of younger and older transgender individuals, as well as their lived experience, may be markedly different.

## 5. Conclusions

Findings from this large survey have highlighted a pressing need for training of health professionals in TGD cultural awareness and health needs. More gender services to meet the demand for TGD health services and in time, mainstreaming of TGD health services in primary care will likely improve accessibility to necessary health services for the TGD community. Furthermore, health service providers should also be mindful of shifting the focus when relevant to highlight the pride and positive experiences of gender diversity. Reliable health resources would complement individualised care provided by health professionals. Further research into optimal models of care and evaluation of training strategies are much needed; however, until further data is available, the community views described should be incorporated into TGD health service delivery and guide research priorities.

## Figures and Tables

**Table 1:** Codebook for survey question: How do you think healthcare professionals can better support you?

Theme	Code
Provide Accessible Care	Improve accessibility to healthcare
	Information for the community
Training and Professional Development	Training for medical professionals
	Professional development
	Communication with the community
	Need for more guidelines, research, advocacy
	Positive experiences

**Table 2:** Codebook for survey question: What do you think are the two most important issues for your health, i.e., if you could improve your health right now, what would you do?

Theme	Code
Accessibility and Training for Healthcare Professionals	Accessibility issues
	Educate health professionals
	Community building, information for the community
Transgender-related medical intervention	Trans and related medical intervention
Health, Wellbeing and Support	General well-being
	Housing, employment, family issues
	Less social discrimination, more social or community support
	Mental well-being
	Other medical issues
	Substance use

**Table 3:** Codebook for survey question: Are there any areas of trans-related medical research that you would like addressed?

Theme	Code
Trans Medical Advancements	Surgical techniques
	Potential associated health issues
	Hormone effects and risks
	Alternative and associated treatment
Accessibility and Standards of Care	Accessibility
	Diagnosis or treatment guidelines
	Community support and communication
Greater Breadth of Research and Understanding	Adolescent issues
	Adult trans research
	Aged care
	All or others
	Mental health, Potential Neurodiversity association
